# The direct healthcare costs attributable to West Nile virus illness in Ontario, Canada: a population-based cohort study using laboratory and health administrative data

**DOI:** 10.1186/s12879-019-4596-9

**Published:** 2019-12-17

**Authors:** Emily Shing, John Wang, Mark P. Nelder, Camilla Parpia, Jonathan B. Gubbay, Mark Loeb, Erik Kristjanson, Alex Marchand-Austin, Stephen Moore, Curtis Russell, Doug Sider, Beate Sander

**Affiliations:** 1 Public Health Ontario, Toronto, Ontario Canada; 2 Institute of Health Policy, Management and Evaluation, University of Toronto, Toronto, Ontario Canada; 3 ICES, Toronto, Ontario Canada; 4 Department of Pathology and Molecular Medicine; Department of Health Research, Evidence, and Impact; Michael G. DeGroote Institute for Infectious Disease Research, McMaster University, Hamilton, Ontario Canada; 5 Toronto Health Economics and Technology Assessment (THETA) Collaborative, University Health Network, Toronto, Ontario Canada

**Keywords:** Healthcare cost, West Nile virus, Cost analysis, Cohort study

## Abstract

**Background:**

West Nile virus (WNV) is a mosquito-borne flavivirus, first detected in the Western Hemisphere in 1999 and spread across North America over the next decade. Though endemic in the most populous areas of North America, few studies have estimated the healthcare costs associated with WNV. The objective of this study was to determine direct healthcare costs attributable to WNV illness in Ontario, Canada.

**Methods:**

We conducted a cost-of-illness study on incident laboratory confirmed and probable WNV infected subjects identified from the provincial laboratory database from Jan 1, 2002 through Dec 31, 2012. Infected subjects were linked to health administrative data and matched to uninfected subjects. We used phase-of-care methods to calculate costs for 3 phases of illness: acute infection, continuing care, and final care prior to death. Mean 10-day attributable costs were reported in 2014 Canadian dollars, per capita. Sensitivity analysis was conducted to test the impact of WNV neurologic syndromes on healthcare costs.

**Results:**

One thousand five hundred fifty-one laboratory confirmed and probable WNV infected subjects were ascertained; 1540 (99.3%) were matched to uninfected subjects. Mean age of WNV infected subjects was 49.1 ± 18.4 years, 50.5% were female. Mean costs attributable to WNV were $1177 (95% CI: $1001, $1352) for acute infection, $180 (95% CI: $122, $238) for continuing care, $11,614 (95% CI: $5916, $17,313) for final care - acute death, and $3199 (95% CI: $1770, $4627) for final care - late death. Expected 1-year costs were $13,648, adjusted for survival. Three hundred seventeen infected subjects were diagnosed with at least one neurologic syndrome and greatest healthcare costs in acute infection were associated with encephalitis ($4710, 95% CI: $3770, $5650).

**Conclusions:**

WNV is associated with increased healthcare resource utilization across all phases of care. High-quality studies are needed to understand the health system impact of vector-borne diseases and evaluate the cost effectiveness of novel WNV interventions.

## Background

West Nile virus (WNV) is a mosquito-borne flavivirus, detected in the Western Hemisphere in 1999 that spread across North America over the next decade [1]. In Canada, WNV was identified in birds and mosquitoes in 2001, followed by the first human cases in 2002 in Ontario and Quebec [2]. From 2002 through 2012, 1039 confirmed and probable cases of WNV were reported to the provincial disease surveillance system in Ontario, with the highest incidence rate of 2.6 cases per 100,000 population in 2002 [3].

Approximately 20% of WNV infections are symptomatic [4]. Commonly referred to as West Nile fever (WNF), symptoms of infection may include fever as well as headache, joint pain, and vomiting; body aches and muscle weakness may persist for months [5, 6]. West Nile neuroinvasive disease (WNND) occurs in 1 in 150 of infections, presenting as meningitis, encephalitis or acute flaccid paralysis (AFP) [7–10]. The case fatality of recent WNV epidemics has varied, ranging from 4 to 14% [11, 12]. Advanced age has been consistently identified as a risk factor for death, with those above 65 years and with comorbid conditions at particularly high risk [6, 13, 14].

Though endemic in the most populous areas of North America, few studies have estimated the healthcare costs associated with WNV [15–18]. Among the literature from the US, cases infected with WNV during a single season have been analyzed to obtain cost estimates. These estimates have ranged largely between epidemics and among varying levels of WNV severity. The median cost of inpatient treatment was estimated to be $8274 USD per patient in Louisiana in 2002 and $25,117 in 2012 in Colorado, where persons diagnosed with AFP incurred the highest costs related to WNV [15–17]. Notably, no literature in Canada has been published on the healthcare costs attributable to WNV illness in Canada. Therefore, using laboratory and health administrative data, the objective of this study is to determine the incidence-based healthcare costs attributable to WNV from the healthcare payer perspective in Ontario, Canada.

## Methods

### Design, data sources, index date, and matched cohort

We conducted an incidence-based matched cohort study to estimate costs attributable to WNV in Ontario, Canada from the healthcare payer perspective (the Ontario Ministry of Health). This study was approved by the Ontario Agency for Health Protection and Promotion (Public Health Ontario) institutional Ethics Review Board. Data analysis was performed using SAS 9.3 (SAS Institute, Cary, NC).

A cohort of incident infected subjects was established from all laboratory confirmed and probable cases of WNV illness from January 1, 2002 through December 31, 2012 from Public Health Ontario Laboratory (PHOL) data by applying the laboratory case definitions [19]. This WNV dataset was linked to health administrative data routinely collected from Ontario’s publicly funded single-payer healthcare system. These datasets were linked using unique encoded identifiers and analyzed at ICES, an independent, non-profit research institute whose legal status under Ontario’s health information privacy law allows it to collect and analyze health care and demographic data, without consent, for health system evaluation and improvement. To estimate an index date for the start of healthcare resource use, we conducted Joinpoint analysis [20] by plotting mean total daily costs 360-days prior to the earliest laboratory confirmation date to observe increasing healthcare costs prior to laboratory confirmation, suggestive of symptom onset. Using this method, the index date for healthcare resource use was assigned as 14 days prior to laboratory confirmation date, taking into account the results of the Joinpoint analysis and expert opinion that the typical incubation period for WNV is 1 to 2 weeks.

Each infected subject was matched to three uninfected subjects from the general population on age ± 1 year, sex, ±30 days index date for healthcare resource use (randomly assigned date ±30 days from the index date for the infected), and propensity score (Table 1). To balance confounder distribution among the infected and uninfected groups, the propensity score regressed infection status (infected versus uninfected) on rurality (using the Rurality Index of Ontario, accessibility to healthcare, geographical measure [21], neighborhood income quintile as a proxy for socioeconomic status, comorbidities ≤2 years prior to index date (using the Johns Hopkins Collapsed Aggregated Diagnosis Groups; CADGs [22].

**Table 1:** Variables used for matching WNV infected subjects to uninfected subjects

	Index date	Death date
*Hard-matched variables*		
Index date	±30 days	N/A
Death date	N/A	±90 days
Age	±1 year	±5 years
Sex	Exact	Exact
*Propensity score variables*		
Rurality Index of Ontario (RIO)	At index date	180 days prior to death
Neighbourhood income quintile	At index date	180 days prior to death
Johns Hopkins ACG® System - collapsed aggregated diagnosis groups (CADG)	2 years prior to index date	180 days prior to death

Abbreviations: Collapsed Aggregated Diagnosis Groups (CADG); Rurality Index of Ontario, RIO;

Each infected subject who died was rematched to three uninfected subjects who also died during the observation period to estimate the impact of WNV on healthcare costs prior to the time of death. Subjects were hard matched on age ± 5 years, sex, and ± 90 days of death date (Table 1). The propensity score regressed infection status on rurality, neighborhood income quintile, and comorbidities 180 days before death date.

Standardized differences were calculated to quantify balance of all variables used in matching. A threshold of 0.1 was used to represent good balance and negligible differences between the infected and uninfected subjects [23].

### Outcomes

Phase-specific attributable costs for the acute and continuing phases of illness were calculated using phase-of-care methods [24, 25]. Three phases were defined by observing the mean daily costs of all infected subjects 360 days after the index date. Three phases were defined as 1) Acute infection – first 90 days after index date, 2) Continuing care, and 3) Final care prior to end of observation period (or death). Final care was further differentiated into two phases: acute death (if the subject died within 30 days after the index date) and late death (if the subject died > 30 days after the index date; phase length 100 days). Attributable costs were calculated as the mean difference between matched infected and uninfected subjects with 95% confidence intervals.

Each subject’s observation time was allocated to each phase in the following order: final care, acute infection, with the remaining time of observation assigned to continuing care. For example, for a subject with 360 days of observation time (index date through death date), the last 100 days would be allocated as final care, the first 90 days would be acute infection, and the remaining time (270 days) would be allocated as continuing care. For subjects observed for less than 90 days (index date to death date was less than the acute infection phase length), the entire observation period would be allocated as final care.

Costs were calculated using person-level costing methods established by ICES [26]. All direct, publicly funded healthcare service categories were included as recommended by the Canadian Agency for Drugs and Technologies in Health. Person-level data across a wide variety of healthcare settings are collected and stored in administrative databases by the Canadian Institute for Health Information (CIHI) and the Ontario Ministry of Health. Through these databases, accountability and efficiency across the entire spectrum of the healthcare system are monitored. In-depth information on all the health administrative databases is provided in the costing guidelines by Wodchis et al. [26].

Fees associated with distinct healthcare service categories were used to derive stratified costs by utilization (e.g., inpatient hospitalization, emergency department visits, physician services; Table 2). Cost outcomes also included expected cumulative costs adjusted for survival, 1 year and 2 years after the index date.

**Table 2:** Healthcare resource utilization categories

Healthcare utilization category	
Acute inpatient hospitalization	
Emergency department visits	
Physician services	
Same day surgeries, other ambulatory treatments (e.g., dialysis, oncology)	
Prescription medication	
Other:	*Inpatient rehabilitation*
	*Inpatient mental health services*
	*Complex continuing care*
	*Long-term care*
	*Home care*
	*Equipment (devices)*

Mean 10-day costs were reported in 2014 Canadian dollars, per capita. Relative risks (RR) for hospitalization after the index date and mortality were estimated using a modified Poisson regression with robust error variances [27] and adjusted for age and sex.

### Sensitivity analysis

To test the impact of severe WNV syndromes on healthcare costs, costs and RRs for hospitalization and mortality were estimated for individuals with WNND syndromes (i.e., meningitis, encephalitis, AFP).

Neurologic syndromes were identified from ICD-10-CA (International Statistical Classification of Diseases and Related Health Problems, 10th Revision, Canada) diagnostic codes and Ontario Health Insurance Plan (OHIP) codes used by physicians for billing in the province of Ontario ±30 days from index date, with input from clinical and WNV content experts. If a subject was diagnosed with > 1 neurologic syndrome, they were classified to the more severe clinical syndrome (AFP > encephalitis > meningitis).

## Results

### Study cohort

From 2002 through 2012, 1551 incident, laboratory confirmed and probable WNV infected subjects were identified from the PHOL dataset and individually linked to health administrative data. 1540 (99.3%) infected subjects were matched to uninfected subjects. Balance was assessed and all standardized differences were below 0.10, indicating good balance (Additional file 2: Table S1). Mean age of WNV infected subjects was 49.1 ± 18.4 years, 778 (50.5%) were female and 61 (4.0%) resided in rural areas in Ontario (Table 3). The median CADG score was 6.0 and 360 (23.4%) of infected patients were among the highest income quintile. Unmatched infected subjects (*n* = 11) were younger than matched, infected subjects (mean age, 40.1 ± 14.8 years) and more likely to reside rurally.

**Table 3:** Demographic descriptive statistics of WNV infected subjects

Variable	WNV infected N (%)	WNV infected, WNND N (%)	WNV infected, non-WNND N (%)
Subjects	1540 (100)	317 (100)	1223 (100)
Mean age at index date, years ± SD	49.1 ± 18.4	53.5 ± 20.9	47.3 ± 17.5
< 5	26 (1.7)	6 (1.9)	20 (1.6)
5–24	130 (8.4)	29 (9.1)	102 (8.3)
25–44	444 (28.8)	60 (18.9)	385 (31.5)
45–54	339 (22.0)	51 (16.1)	287 (23.5)
55–64	303 (19.7)	65 (20.5)	239 (19.5)
65–74	178 (11.6)	54 (17.0)	122 (10.0)
75–84	104 (6.8)	44 (13.9)	60 (4.9)
≥85	16 (1.0)	8 (2.5)	8 (0.7)
Sex, female	778 (50.5)	128 (40.4)	650 (53.1)
Rural residence			
Rural	61 (4.0)	<10	46 (3.8)
Non-rural	1479 (96.0)	306 (96.5)	1177 (96.2)
Missing		≤5	
Income quintile			
1 (lowest)	235 (15.3)	45 (14.2)	198 (16.2)
2	312 (20.3)	67 (21.1)	227 (18.6)
3	319 (20.7)	70 (22.1)	265 (21.7)
4	314 (20.4)	61 (19.2)	235 (19.2)
5 (highest)	360 (23.4)	74 (23.3)	297 (24.3)

Abbreviations: standard deviation, SD, West Nile neuroinvasive disease, WNND; West Nile virus, WNV

Among infected subjects who died, 162 (99.4%) subjects were each matched to three uninfected subjects who also died. Mean age at death was 70.9 ± 14.5 years, 104 (64.2%) were male, and 3 (1.9%) resided rurally. Nearly all standardized differences were below 0.10 indicating good balance between matched infected and uninfected subjects who died.

**Fig. 1:**
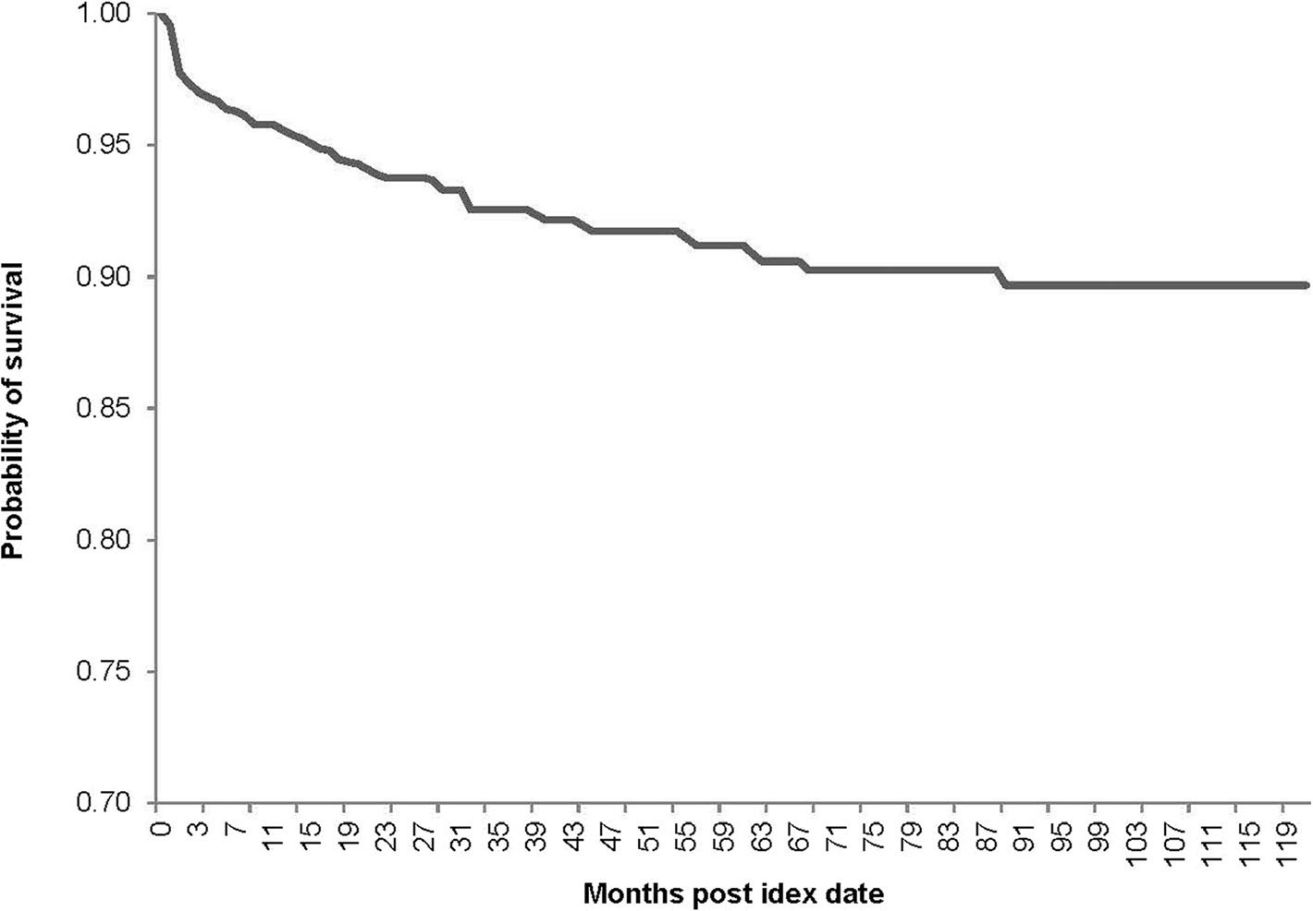
Survival curve of WNV infected subjects. Probability of survival from date of index infection over 10 years. Among WNV infected subjects, 24 (1.5%) died within 1month of index date, 72 (4.6%) died within 1 year of index date, and 163 (10.5%) died throughout the entire observation period

Median duration of follow-up for infected subjects was 3.6 years. 547 (35.3%) subjects were admitted to hospital within 30 days of index date (mean length of stay was 17.7 ± 25.7 days; Table 4). Among infected subjects, all-cause mortality was 10.5%. All-cause mortality 1 year after index date was 4.6% (Table 4). The 10-year survival curve for WNV infected subjects is depicted in Fig 1.

**Table 4:** Descriptive statistics of WNV clinical outcomes

	N (%)		
	WNV	WNND	Non-WNND
*Subjects*	1540	317	1223
*Hospital admission 5 days after index date*	77 (5.0)	39 (12.3)	39 (2.9)
Length of stay, mean days ± SD	20.8 ± 28.4	28.5 ± 27.1	11.5 ± 27.8
*Hospital admission 30 days after index date*	547 (35.3)	273 (86.1)	271 (22.2)
Length of stay, mean days ± SD	17.7 ± 25.7	22.3 ± 27.0	12.9 ± 23.4
Mortality			
10-day	7 (0.4)	≤5	<10
30-day	24 (1.5)	10 (3.2)	14 (1.1)
90-day	45 (2.9)	19 (6.0)	26 (2.1)
1-year	72 (4.6)	29 (9.1)	43 (3.5)
*Relative Risks (95% CI)*			
WNV vs. uninfected, hospitalization 30 days after index date		1.0 (0.99, 1.01)	
WNV vs. uninfected, all-cause mortality		1.0 (0.99, 1.01)	
*Sensitivity Analysis*			
WNND vs. non-WNND, hospitalization 30 days post-index		6.4 (5.1, 8.0)	
WNND vs. non-WNND, all-cause mortality		1.9 (1.5, 2.3)	

Abbreviations: confidence interval, CI; standard deviation, SD; West Nile neurologic disease, WNND West Nile virus, WNV

### Cost outcomes

**Fig. 2:**
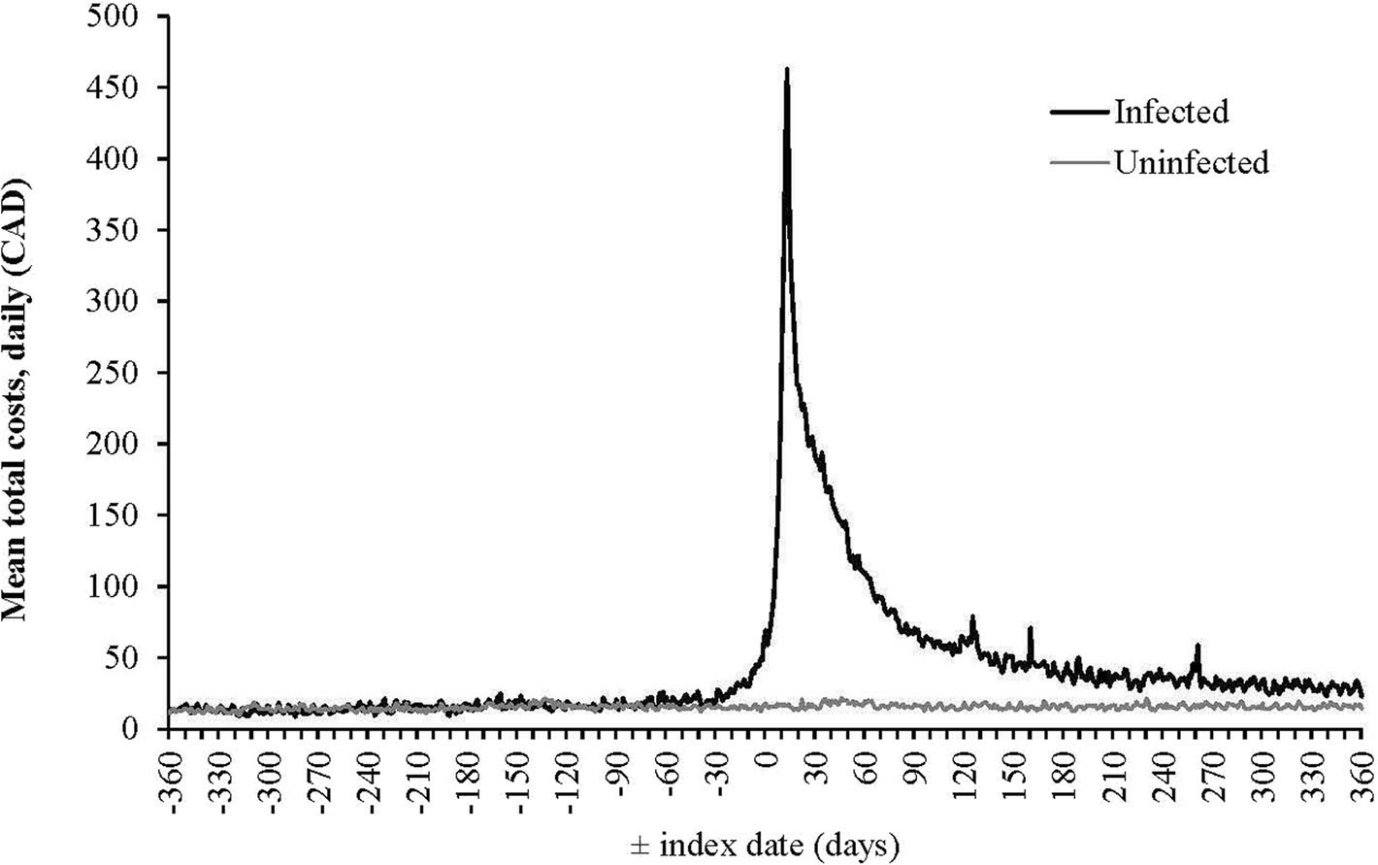
Mean total costs 360 days pre- and post-index date of WNV infected and uninfected subjects

**Fig. 3:**
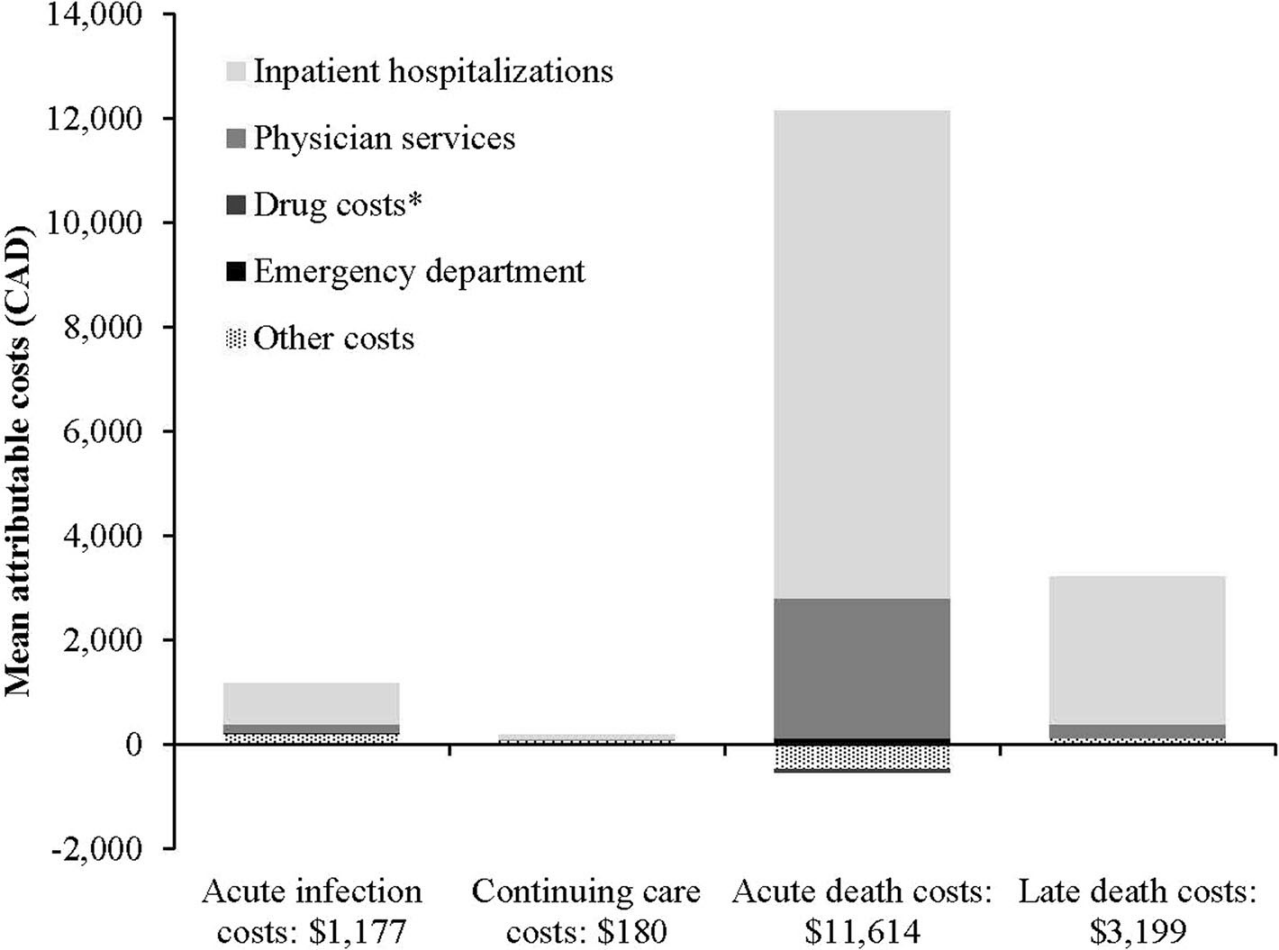
Phase-specific WNV attributable costs for all infected subjects, stratified by healthcare services. Acute infection (n = 1,495), continuing care (n = 1,485), acute death (n = 24), late death (n = 129)

Costs exhibited a “U” shaped curve from WNV index date to death (Additional file 1: Figure S1) and costs 360 days pre- and post-index date of matched WNV infected and uninfected subjects are show in Fig. 2. Mean costs attributable to WNV were $1177 (95% CI: $1001, $1352) for acute infection, $180 (95% CI: $122, $238) for continuing care, $11,614 (95% CI: $5916, $17,313) for final care - acute death, and $3199 (95% CI: $1770, $4627) for final care - late death (Table 5). Hospitalization costs in the acute infection phase were $781 (95% CI: $636, $927), followed by other costs ($197, 95% CI: $150, $244), and physician services costs ($161, 95% CI: $143, $180) (Fig. 3).

**Table 5:** Cost outcomes attributable to WNV illness, by phase of illness

	N	WNV Infected	Uninfected	Attributable cost (95% CI)
All WNV infected subjects				
*Mean cost outcomes by phase*				
Acute infection	1495	$1277	$101	$1177 ($1001, $1352)
Continuing care	1485	$283	$103	$180 ($122, $238)
Final care - acute death	24	$16,464	$4850	$11,614 ($5916, $17313)
Final care - late death	129	$6369	$3170	$3199 ($1770, $4627)

Abbreviations: confidence interval, CI; West Nile neurologic disease, WNND; West Nile virus, WNV

Greatest costs were associated with healthcare resources utilized from inpatient hospitalization and physician services in final care - acute death. Average inpatient hospitalization costs during this phase were $9355, 95% CI: $4921, $13,789; physician services costs were $2671, 95% CI: $537, $4804. Final care - late death costs were primarily inpatient hospitalization costs ($2823, 95% CI: $1451, $4196).

Expected cumulative mean attributable one-year and two-year costs were $13,648 and $20,124, respectively, adjusted for survival. No significant difference for RR of hospitalization within 30 days after the index date or all-cause mortality between WNV infected compared to uninfected subjects was observed (1.0, 95% CI: 0.99, 1.01) (Table 4).

### Sensitivity analysis

A total of 317 infected subjects were diagnosed with at least one WNND syndrome; mean age of subjects was 53.5 ± 20.9 years (Table 3).

Mean attributable costs for acute infection and continuing care were greater for WNV infected subjects with WNND syndromes compared to non-WNND subjects. Acute infection costs for WNND subjects were $3576 (95% CI: $2999, $4151), $507 (95% CI: $263, $751) for continuing care, $4588 (95% CI: -$957, $10,133) for final care - acute death, $5164 (95% CI: $2411, $7917) for final care - late death (Table 6).

**Table 6:** Cost outcomes attributable to WNND, by phase of illness

WNND	N	WNND	Non-WNND	Attributable cost (95% CI)
Acute infection	294	$3688	$114	$3576 ($2999, $4151)
Continuing care	291	$636	$129	$507 ($263, $751)
Final care - acute death	10	$11,762	$7174	$4588 (−$957, $10,133)
Final care - late death	54	$7596	$2433	$5164 ($2411, $7917)
WNND, encephalitis		Encephalitis	Non-WNND	Attributable cost (95% CI)
Acute infection	141	$4820	$110	$4710 ($3770, $5650)
Continuing care	139	$1065	$150	$915 ($422, $1408)
Final care - acute death	9	$12,545	$7328	$5216 (−$807, $11,239)
Final care - late death	42	$8599	$2400	$6199 ($2850, $9547)
WNND, AFP		AFP	Non-WNND	Attributable cost (95% CI)
Acute infection	74	$3499	$115	$3384 ($2247, $4521)
Continuing care	74	$387	$119	$267 ($109, $426)
Final care - acute death	NR	NR	NR	NR
Final care - late death	8	$3707	$2920	$787 (−$2648, $4223)
WNND, meningitis		Meningitis	Non-WNND	Attributable cost (95% CI)
Acute infection	79	$1847	$119	$1728 ($1126, $2329)
Continuing care	78	$108	$101	$6 (−$50, $62)
Final care - acute death	10	$11,762	$7174	$4588 (−$957, $10,133)
Final care - late death	NR	NR	NR	NR
WNV infected, excluding WNND		Non-WNND	Uninfected	Attributable cost (95% CI)
Acute infection	1192	$623	$95	$527 ($399, $656)
Continuing care	1185	$194	$96	$99 ($55, $142)
Final care - acute death	14	$19,822	$4648	$15,175 ($6154, $24,195)
Final care - late death	77	$4578	$2475	$2103 ($700, $3506)

**Fig. 4:**
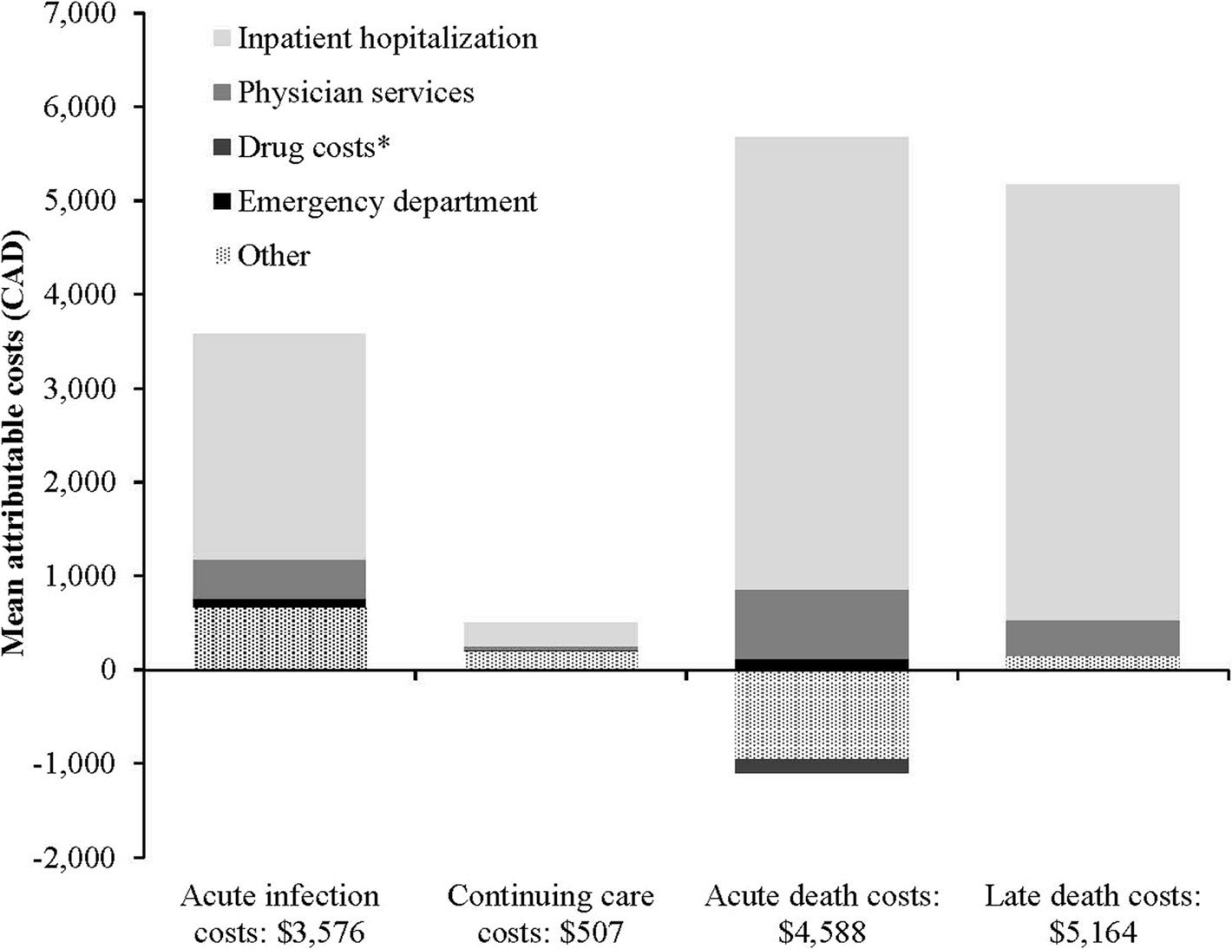
Phase-specific attributable costs for WNV infected subjects with WNND syndromes only, stratified by healthcare services

Hospitalization costs were the predominant cost component across every phase of care. In acute infection, hospitalization costs were $2392 (95% CI: $1919, $2864), $254 (95% CI: $55, $452) in continuing care, $4827 (95% CI: -$597, $10,251) in final care - acute death, and $4639 (95% CI: $2005, $7273) in final care - late death (Fig. 4).

In acute infection, greatest healthcare costs were associated with encephalitis ($4710, 95% CI: $3770, $5650), followed by AFP ($3384, 95% CI: $2247, $4521), and then meningitis ($1728, 95% CI: $1126, $2329) (Table 6). During the continuing care phase, mean attributable costs for subjects with encephalitis were $915 (95% CI: $422, $1408), whereas continuing care costs for infected subjects with meningitis were not different from uninfected matched subjects ($6, 95% CI: -$50, $62). Among non-WNND infected subjects, acute infection costs were $527 (95% CI: $399, $656) and continuing care costs were $99 (95% CI: $55, $142).

RR of hospitalization within 30 days after the index date was 6.4 (95% CI: 5.1, 8.0) for those with WNND syndromes compared to non-WNND infected subjects, adjusted for age and sex. Adjusted RR for all-cause mortality was 1.9 (95% CI: 1.5, 2.3) for infected subjects with WNND syndromes compared to non-WNND infected subjects (Table 4).

## Discussion

The aim of this study was to estimate the direct healthcare costs attributable to incident WNV infection in Ontario from 2002 through 2012. We found each infected subject incurred $13,648 in direct healthcare costs over 1 year attributable to WNV, adjusted for survival. Inpatient hospitalizations contributed the greatest costs across all phases of care and patients with encephalitis incurred the greatest costs among those with WNND. The risk of mortality and hospitalization was significantly greater among infected subjects with WNND syndromes compared to non-WNND infected subjects. Estimating costs associated with loss of productivity and costs attributable to WNV sequelae were beyond the scope of this study.

While no previous studies have been conducted in Canada to estimate healthcare costs attributable to WNV, studies in the US have reported that WNV epidemics are a substantial economic burden on healthcare resources [16, 17]. Barber et al., found that outpatient healthcare costs were $6317 and inpatient healthcare costs were $33,143 to treat WNV during a 2005 outbreak [16]. Staples et al., estimated initial hospitalization costs of 80 patients hospitalized with WNND in 2003 and found initial costs were highest for patients with AFP (median $20,774) and encephalitis ($15,136), whereas meningitis was associated with $7261 and WNF was associated with $4467 [15]. Using data from the Louisiana epidemic in 2002, Zohrabian et al. found the median cost per hospitalized patient was $8274 [17]. Each US study found healthcare required to treat cases of WNV was considerable, however, did not use matched cohort methods to determine the costs attributable to WNV. To contextualize the costs of WNV with another prevalent vector-borne disease in Ontario, the costs attributable to Lyme disease were estimated to be $832 over 1 year in Ontario, Canada [28] and $3048 over 1 year in the US [29], whereas the present study estimated the costs attributable to WNV in 1 year to be $13,648. This magnitude of difference in attributable healthcare costs highlights the variability of healthcare resource utilization between vector-borne diseases. Using Canadian data and rigorous case-costing methods to determine the healthcare utilization of WNV infected subjects compared to uninfected subjects, our study is a complement to the published literature and further illustrates the magnitude of healthcare costs associated with WNV.

Though epidemiologic studies have estimated that 1 in 150 WNV infections results in WNND [10], 20% of the subjects in our WNV cohort had at least one diagnostic code for neurologic syndromes ±30 days of their index date. Therefore, our estimation of the costs attributable to each case of WNV and the overall economic burden of WNV on the healthcare system is likely an overestimation of the average WNV infection prognosis as typically only a small proportion of infected subjects would develop WNND or require hospitalization [30]. Nonetheless, using a large cohort of laboratory-confirmed and probable WNV infected subjects, a strength of our study lies in the specificity of the subjects included. By ascertaining an infected cohort from the provincial laboratory database, all subjects included in our study were diagnosed on the basis of laboratory confirmation. In addition, applying a matched study design to reduce confounding, and linking laboratory and health administrative databases to estimate costs from the healthcare payer perspective are further strengths of this study.

To test the sensitivity of WNND syndromes on costs, we conducted a sensitivity analysis to estimate the costs associated with and without neurologic syndromes. The analysis found acute infection and continuing care costs were greater for those with WNND syndromes (costs for encephalitis > AFP > meningitis) compared to the remaining subjects with non-WNND infections. Due to the low incidence of AFP syndromes, we were unable to draw reliable estimates on the attributable mortality costs of patients with AFP. Notably, previous case reports on patients with AFP from WNV infections found physical recovery was incomplete after 1 year of follow-up, suggesting the potential for long-term costs associated with WNV sequelae [31, 32]. For our sensitivity analysis, we were able to identify infected subjects with neurologic syndromes from diagnostic and billing codes, however, we assumed that in the absence of any of the neurologic codes the infection could be considered as non-WNND. Future studies may investigate further both the economic burden of these infections since they may be prone WNV sequelae and incur significant long-term costs on the healthcare system [6].

## Conclusion

In conclusion, by applying population-based methods to estimate the costs of WNV from the healthcare payer perspective, we found that healthcare resource utilization is elevated across all phases of care attributable to WNV infections and WNND syndromes. Though current evidence supports that WNND results rarely from WNV infections, a large proportion of WNV infected individuals who interface with the healthcare system in Ontario have severe neurologic conditions temporally associated with WNV infection, requiring increased healthcare resource use predominantly from hospitalization and physician services. High costs of treatment underscore the importance of evaluating the cost effectiveness of WNV and other mosquito-borne disease prevention through vector control strategies (e.g., draining surface water, larviciding) and novel vaccinations to inform healthcare resource allocation in Ontario.

## Supplementary information

**Additional file 1: Figure S1.** Panel A shows the mean total costs of WNV infected subjects (*n* = 1551) 360 days after index date. Panel B shows the mean total cost of all WNV infected subjects who died during the observation period (*n* = 163)

**Additional file 2: Table S1.** Baseline characteristics of WNV infected subjects matched ±30 days from index date (*n* = 1540)

## Data Availability

The dataset from this study is held securely in coded form at ICES. While data sharing agreements prohibit ICES from making the dataset publicly available, access may be granted to those who meet pre-specified criteria for confidential access, available at www.ices.on.ca/DAS. The full dataset creation plan and underlying analytic code are available from the authors upon request, understanding that the computer programs may rely upon coding templates or macros that are unique to ICES and are therefore either inaccessible or may require modification.

## Abbreviations

AFP: acute flaccid paralysis
CADGs: Johns Hopkins Collapsed Aggregated Diagnosis Groups
CIHI: Canadian Institute for Health Information
ICD-10-CA: International Statistical Classification of Diseases and Related Health Problems, 10th Revision, Canada
OHIP: Ontario Health Insurance Plan
PHOL: Public Health Ontario Laboratory
RR: Relative risks
WNF: West Nile fever
WNND: West Nile neuroinvasive disease
WNV: West Nile virus
