# Supplementary figures and images for: The direct healthcare costs attributable to West Nile virus illness in Ontario, Canada: a population-based cohort study using laboratory and health administrative data
Source: BMC Infect Dis. 2019 Dec 17;19:1059. doi: 10.1186/s12879-019-4596-9 (PMC6918579; doi:10.1186/s12879-019-4596-9)

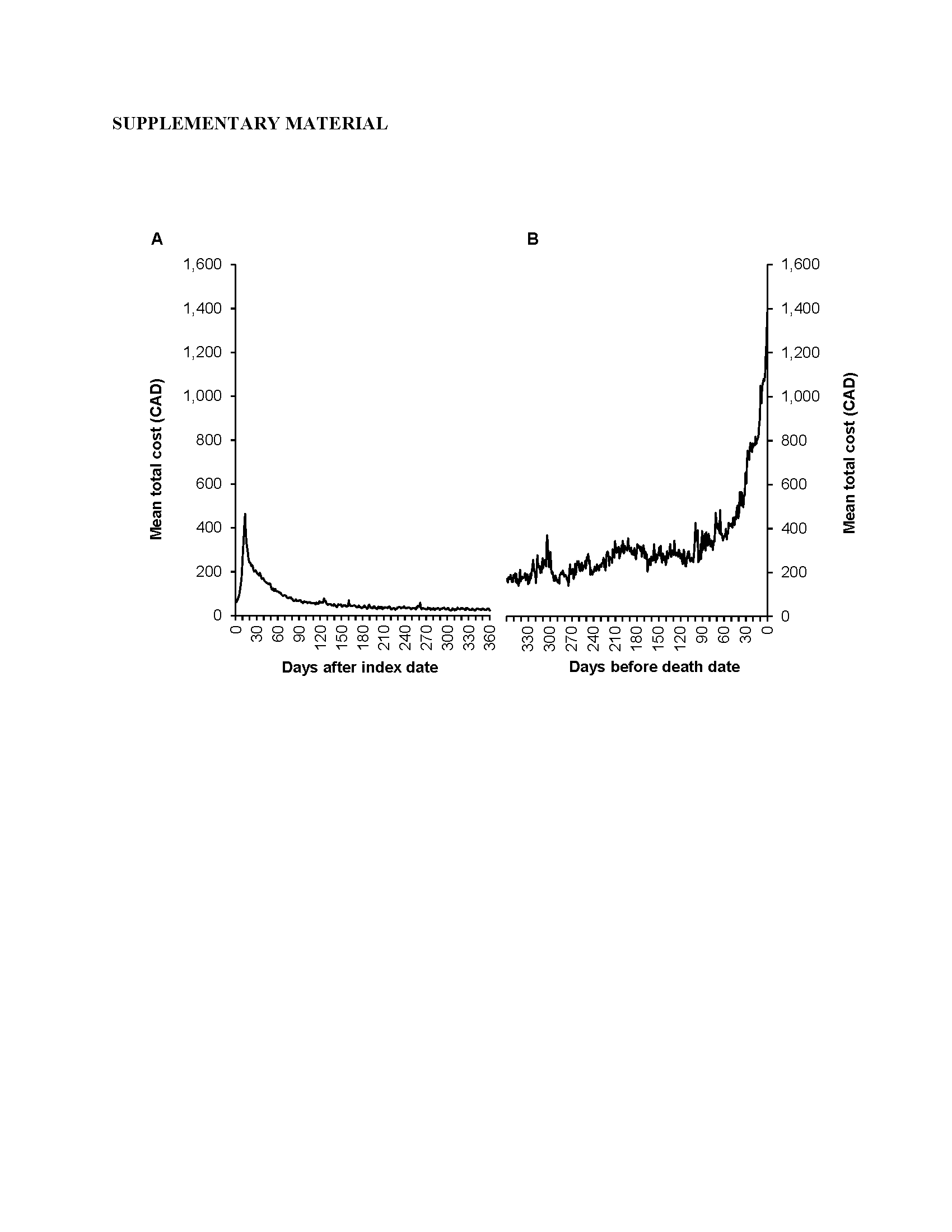

Supplement: Supplementary file 1 — Additional file 1: Figure S1. Panel A shows the mean total costs of WNV infected subjects (n = 1551) 360 days after index date. Panel B shows the mean total cost of all WNV infected subjects who died during the observation period (n = 163) [file 12879_2019_4596_MOESM1_ESM.tif]
