# Supplementary material for: The direct healthcare costs attributable to West Nile virus illness in Ontario, Canada: a population-based cohort study using laboratory and health administrative data
Source: BMC Infect Dis. 2019 Dec 17;19:1059. doi: 10.1186/s12879-019-4596-9 (PMC6918579; doi:10.1186/s12879-019-4596-9)
Supplement: Supplementary file 2 — Additional file 2: Table S1. Baseline characteristics of WNV infected subjects matched ±30 days from index date (n = 1540) [file 12879_2019_4596_MOESM2_ESM.docx]

| Characteristic | Exposed | Unexposed | SD |
| --- | --- | --- | --- |
| Subjects | 1,540 | 4,620 | -- |
| Age at index date, years | 49.06 ± 18.4 | 49.07 ± 18.4 | 0.001 |
| Sex |  |  |  |
| Male | 0.49 | 0.49 | 0 |
| Female | 0.51 | 0.51 | 0 |
| Propensity score | 8.68 | 8.68 | 0.001 |
| Rurality |  |  |  |
| Non-rural 0-39 | 0.96 | 0.95 | 0.033 |
| Rural ≥40 | 0.04 | 0.05 | 0.033 |
| CADG |  |  |  |
| Mean Score | 6.44 | 6.09 | 0.095 |
| Income quintile |  |  |  |
| 1 (low) | 0.15 | 0.15 | 0.001 |
| 2 | 0.20 | 0.20 | 0.004 |
| 3 | 0.21 | 0.20 | 0.008 |
| 4 | 0.20 | 0.20 | 0.003 |
| 5 (high) | 0.23 | 0.24 | 0.008 |

**Table S1**. Baseline characteristics of WNV infected subjects matched ±30 days from index date.
